# Comparing depression, anxiety, and quality of life in individuals with cardiac and non-cardiac chest pain

**DOI:** 10.3389/fpsyt.2023.1302715

**Published:** 2024-01-16

**Authors:** Elham Zarean, Zahra Bahrami Samani, Soleiman Kheiri, Samaneh Torkian

**Affiliations:** ^1^Department of Psychiatry, School of Medicine, Shahrekord University of Medical Sciences, Shahrekord, Iran; ^2^Clinical Research Development Unit, Hajar Hospital, Shahrekord University of Medical Sciences, Shahrekord, Iran; ^3^Department of Epidemiology and Biostatistics, School of Public Health, Shahrekord University of Medical Sciences, Shahrekord, Iran; ^4^Department of Epidemiology, School of Public Health, Iran University of Medical Sciences, Tehran, Iran

**Keywords:** anxiety, cardiac, chest pain, depression, non-cardiac, quality

## Abstract

**Background:**

Psychological factors are often overlooked as potential contributors to cardiovascular disease. This study aimed to investigate the relationship between depression, anxiety, and quality of life with chest pain origin.

**Method:**

This cross-sectional study was performed from 2019 to 2020 and included participants from multiple medical centers across Shahrekord, Iran. Participants were recruited through advertisements in medical centers. Participants were divided into three groups: healthy control (*n* = 67), chest pain with cardiac origin (CCP) (*n* = 70), and chest pain with non-cardiac origin (NCCP) (*n* = 73). Data were collected using the Beck’s Anxiety scale, Beck’s Depression scale, and Short-Form Health Survey questionnaires. The chi-square, exact test, *t*-test, Kruskal–Wallis, and logistic regression models were used for statistical analysis. All analysis was performed using SPSS 26.

**Results:**

The mean scores of depression and anxiety in the NCCP group (depression = 17.03 ± 11.93, anxiety = 17.18 ± 11.37) were significantly higher than those in the CCP (depression = 9.73 ± 5.76, anxiety = 8.77 ± 5.96) and healthy (depression = 7.00 ± 7.61, anxiety = 6.18 ± 7.63) groups (*p* < 0.05). The mean score of quality of life in the NCCP group (54.87 ± 12.66) was significantly lower than that in the CCP (76.31 ± 12.49) and healthy (80.94 ± 15.78) groups (*p* < 0.05). Patients with NCCP had higher odds of having depression (adjusted OR = 4.39, 95% CI: 1.25, 15.35) and lower odds for having mental quality of life scores than the CCP and health groups, respectively (adjusted OR = 0.90, 95% CI: 0.87, 0.94).

**Conclusion:**

Our findings suggest that collaboration between psychiatrists and other specialists may be necessary to improve patients’ health conditions and quality of life.

## Introduction

Chest pain is one of the most common reasons for attendance at emergency medicine and cardiovascular clinics. The source of chest pain can be cardiac (coronary or non-coronary) and non-cardiac (1). Chest pain in a patient with known coronary artery disease indicates a new or unresolved medical or psychological problem (2). In people with non-cardiac chest pain (NCCP), no cardiac cause for chest pain is found (3). Pulmonary disorders, gastrointestinal disorders, osteoarthritis of the neck, and psychological factors are the most common causes of NCCP (4).

The prevalence of NCCP is up to 70%. NCCP may be diagnosed at all levels of the medical care system, including general practitioners, emergency departments, and chest pain and heart care units (5). Epidemiological studies in different countries such as Germany, Europe, the United States, China, and Australia show that the NCCP prevalence in patients with chest pain is reported to be between 20 and 40% (6). In the United States, cardiac chest pain (CCP) accounts for 38% of emergency department visits, and in only 13% of patients, acute coronary syndrome is identified as the cause of chest pain (7).

There are some evidence that mental health problems are common in patients with chest pain. Anxiety disorders (such as panic disorder and phobia), sleep problems, depressive disorders, poor quality of life (QOL), somatization, and alexithymia are common in patients with chest pain (8, 9). Psychological problems such as depression and anxiety not only have been associated with NCCP but also they are independent risk factors for coronary artery disease (CAD) (3, 10, 11). Furthermore, depression and anxiety can deteriorate the risk factors of CHD, consisting of smoking, high blood pressure, and hyperlipidemia (12, 13). Although 15 to 20% of patients with myocardial infarction were depressed for at least 6 months before the stroke, depression has not been considered in many of these patients (13).

In today’s stressful life, one of the most common manifestations of anxiety and depression can be NCCP. Moreover, psychological problems can worsen the quality of life and sense of wellbeing among patients with either cardiac or non-cardiac chest pain. Numerous studies have examined anxiety, depression, and quality of life in diverse populations (14–17), but there is a lack of research specifically focusing on individuals with chest pain.

Due to the importance of this issue, in this study, we aimed to compare depression, anxiety, and quality of life in patients with chest pain (cardiac and non-cardiac) and healthy individuals referred to cardiac clinics. Studying the effects of chest pain on anxiety, depression, and quality of life in people will provide valuable insight into how chest pain affects individuals across different populations. This information can then be used to develop better treatments for those suffering from chest pain and its associated mental health issues.

## Method

### Design study and participant

This multi-center study examined the relationship between anxiety, depression, and quality of life in people with chest pain. This cross-sectional study was performed from 2019 to 2020 and included participants from multiple medical centers across Shahrekord, Iran. Participants were recruited through advertisements in medical centers. All participants had written consent to enter the study. The questionnaires were completed through face-to-face interviews.

The inclusion criteria for the study were adults aged over 45 years old. We considered adults aged 45 years for simple sample collection because ischemic heart diseases are more common in older people. The inclusion criteria for the NCCP and CCP groups were adults aged over 45 years old who have been diagnosed with chest pain and are currently receiving treatment for their condition. The exclusion criteria included musculoskeletal disorders, gastrointestinal disorders, gastroesophageal reflux disease, pulmonary disorders, heart valve disorders, neck osteoarthritis, and smoking (4, 6). At this stage, we used the restriction method, which is one of the ways to control confounders in the study design stage. We aimed to emphasize psychological factors as a probable causality for NCCP.

Subjects were in three groups: healthy, CCP, and NCCP. Individuals with chest pain (with and without cardiac origin) were selected with the approval of cardiologists and internists. Healthy individuals were recruited among patients referred for other reasons (without chest pain symptoms) based on the inclusion and exclusion criteria.

### Sample size

Considering the Cohen medium effect size of 0.25 (18), three groups (*n*_1_ = *n*_2_ = *n*_3_), 95% confidence level, and 90% power, the total sample size of the study was calculated as 207. G-Power software version 3.1.9.4 was used for this purpose. Finally, 210 individuals were included in the present study. The sampling method was convenient.

### Instruments and variables

Data collection involved a survey administered to participants. The survey assessed anxiety, depression, and quality of life using validated scales. Beck’s Depression Inventory (BDI) (19), Beck’s Anxiety Inventory (BAI) (20), and the Short-Form Health Survey (SF-36) (21) were applied for data collection. Additionally, demographic information such as age, sex, and job were collected.

The BDI contains 21 items, a self-report rating inventory that measures characteristic attitudes and symptoms of depression. The BDI-II contains 21 items on a 4-point scale from 0 (symptom absent) to 3 (severe symptoms). Scoring is achieved by adding the highest ratings for all 21 items. The minimum score is 0, and the maximum score is 63. Higher scores indicate greater symptom severity. In this questionnaire, scores from 0 through 9 indicate no or minimal depression; scores from 10 through 18 indicate mild-to-moderate depression; scores from 19 through 29 indicate moderate-to-severe depression, and scores from 30 through 63 indicate severe depression (19).

The BAI consists of 21 items with a Likert scale ranging from 0 to 3 and raw scores ranging from 0 to 63. The BAI scores are classified as minimal anxiety (0 to 7), mild anxiety (8 to 15), moderate anxiety (16 to 25), and severe anxiety (30 to 63). Each item allows the patient four choices of no symptom to severe symptom. In each item, the patient is asked to report how she/he felt during the past week (20).

The SF-36 Health Survey is a general quality of life instrument that contains 36 questions. It considers the signs of perceived change in health-related quality of life over the past year. Each dimension was the score with a value range of 0–100. A higher score shows a better quality of life. This questionnaire measures two general aspects: physical and mental. The validity and reliability of all questionnaires have already been confirmed in Iran (21–23).

### Analysis

In descriptive analysis, mean, standard deviation (SD), frequency (n), and percentage (%) were used to summarize participant characteristics. The normality of age, depression, anxiety, and physical and mental quality of life was assessed using the Kolmogorov–Smirnov test. Non-parametric tests were used because of the non-normality of the data. In the analytical analysis, chi-square, exact test, t-test, Kruskal–Wallis with Tukey’s HSD *post-hoc*, univariable logistic regression, and multivariable logistic regression were applied to examine the relationship between the source of pain and the psychological variables such as depression, anxiety, and quality of life.

We combined the CCP and healthy groups in logistic models as reference groups. Enter and backward approaches were applied in univariable and multivariable logistic regression models, respectively. We used Lemeshow’s (2000) (24) strategy for assessing potential confounders and interaction forms. In the univariable models, variables with value of ps less or equal to 0.2 were entered into the multivariable model. We examined the interaction between pairs of included risk factors in the multivariable logistic regression model. A variance inflation factor (VIF) was used for checking multicollinearity, which measures the correlation and its strength between the predictor variables in a regression model. The VIF value was less than 10, which means there is no multicollinearity in the independent variables.

In logistic models, job, depression, and anxiety variables were considered binary due to the small sample size in some of their subgroups. The job variable was considered unemployed/housewife and employed, while the employed sub-variable was created from the sum of an employee, freelance job, farmer, worker, and other subscales. The depression and anxiety variables were considered no/mild, minimal/mild, and severe/moderate. All statistical analysis was conducted using SPSS software version 26. A *p*-value of <0.05 was considered statistically significant.

### Ethics

Written informed consent was obtained from all the study participants. The study was conducted in accordance with the Declaration of Helsinki and approved by the Ethics Committee of Shahrekord University of Medical Sciences (IR.SKUMS.REC.1398.032).

## Results

A total of 210 people participated in this study (67 persons were in the healthy group, 70 in the CCP group, and 73 in the NCCP group). In total, 61.9% of participants were women and unemployed/housewives (36.2%). The mean (SD) age of participants was 51.19 (± 6.14) years. Out of the total number of subjects, 4.8% had severe depression and 9% had anxiety. The mean ± SD score of quality of life in the physical and mental areas was 79.54 ± 12.94 and 70.33 ± 17.80, respectively (Table 1). According to chi-square analysis, there is a significant relationship between the pain origin and depression, anxiety, and physical and mental quality of life (*p* < 0.05). The status of demographic and psychological variables has been shown in Table 1.

**Table 1:** Status of demographic variables in healthy, CCP^a^, and NCCP^b^ groups (*n* = 210).

Variables	Total *N* (%)	Healthy (*n* = 67) *N* (%)	Chest pain (*n* = 140)		*p*-value
			CCP (*n* = 70)	NCCP (*n* = 73)	
Age [year (mean ± SD)] Median (IQR)	51.19 ± 6.14 49 (4)	50.52 ± 5.68 49 (4)	50.97 ± 6.20 49 (3.5)	52.00 ± 6.47 49 (4.5)	0.317^c^
Sex					
Female	130 (61.9)	44 (65.7)	44 (62.9)	42 (57.5)	0.600^d^
Male	80 (38.1)	23 (34.3)	26 (37.1)	31 (42.5)	
Job					
Worker	7 (3.3)	0 (0)	5 (7.1)	2 (2.7)	0.202^e^
Farmer	6 (2.9)	1 (1.5)	4 (5.7)	1 (1.4)	
Freelance job	36 (17.2)	10 (14.9)	11 (15.7)	15 (20.5)	
Employee	61 (29.0)	23 (34.3)	16 (22.9)	22 (30.1)	
Unemployed/housewife	76 (36.2)	25 (37.3)	29 (41.4)	22 (30.1)	
Others	24 (11.4)	8 (11.9)	5 (7.1)	11 (15.1)	
Depression					
No	149 (71.0)	63 (91.0)	57 (81.5)	31 (42.5)	<0.001^e^
Mild	30 (14.2)	3 (4.5)	11 (15.7)	16 (21.9)	
Moderate	21 (10.0)	3 (4.5)	1 (1.4)	17 (23.3)	
Severe	10 (4.8)	0 (0.0)	1 (1.4)	9 (12.3)	
Anxiety					
Minimal	101 (48.1)	50 (74.6)	32 (45.7)	101 (48.1)	<0.001^d^
Mild	56 (26.7)	11 (16.4)	29 (41.4)	56 (26.7)	
Moderate	34 (16.2)	3 (4.5)	8 (11.4)	34 (16.2)	
Severe	19 (9.0)	3 (4.5)	1 (1.4)	19 (9.0)	
Quality of life					
Physical	79.54 ± 12.94	87.06 ± 10.30	80.10 ± 13.32	72.08 ± 10.45	<0.001^c^
Mental health	70.33 ± 17.80	80.94 ± 15.78	76.31 ± 12.49	54.87 ± 12.66	

a CCP, cardiac chest pain.

b NCCP, non-cardiac chest pain.

c Kruskal–Wallis test *p*-value.

d Chi-square *p*-value.

e Exact test *p*-value.

Two-by-two groups of pain origin comparison based on the scores of depression, anxiety, and quality of physical and mental life can be seen in Table 2. The median score of depression and anxiety in the NCCP was higher than in the other groups (*p* < 0.05). These scores were higher in CCP compared with the health group (*p* < 0.05). The median score of physical and mental quality of life in the NCCP was much lower than the other two groups (health and CCP) (*p* < 0.05). This score was the highest in the healthy group.

**Table 2:** A two-by-two comparison of healthy, CCP^a^, and NCCP^b^ groups according to psychological variables (*n* = 210).

Variables	Total Mean ± SD Median (IQR: Q3-Q1)	Healthy (*n* = 67) Mean ± SD Median (IQR: Q3-Q1)	Chest pain (*n* = 140)		*p*-value^c^ (CCP & NCCP)	*p*-value^c^ (CCP & Healthy)	*p*-value^c^ (NCCP & Healthy)
			CCP (*n* = 70) Mean ± SD Median (IQR)	NCCP (*n* = 73) Mean ± SD Median (IQR)			
Depression	11.40 ± 9.39 9.00 (15.00–5.00)	7.00 ± 7.61 6.00 (10.00–3.00)	9.73 ± 5.76 9.00 (12.25–6.00)	17.03 ± 11.93 16.00 (23.00–9.00)	<0.001	0.023	<0.001
Anxiety	10.87 ± 9.85 8.00 (16.00–3.75)	6.18 ± 7.63 4 (8.00–2.00)	8.77 ± 5.96 8.00 (12.00–4.00)	17.18 ± 11.37 18.00 (24.00–7.00)	<0.001	0.009	<0.001
Quality of life (Physical)	79.54 ± 12.94 83.12 (89.37–72.50)	87.06 ± 10.30 90.00 (93.75–85.62)	80.10 ± 13.32 84.06 (87.65–77.81)	72.08 ± 10.45 73.12 (77.50–69.68)	<0.001	<0.001	<0.001
Quality of life (Mental)	70.33 ± 17.80 73.97 (85.40–55.05)	80.94 ± 15.78 87.87 (91.00–74.41)	76.31 ± 12.49 80.93 (84.75–71.34)	54.87 ± 12.66 54.66 (60.81–46.54)	0.022	<0.001	<0.001

a CCP, cardiac chest pain.

b NCCP, non-cardiac chest pain.

c Kruskal Wallis test (post-hoc: LSD).

The odds of moderate-to-severe depression and anxiety and quality of life status in the NCCP than CCP/health group are presented in Table 3. After controlling for other potential risk factors, we estimated that the NCCP group had more than four times higher odds of depression than the CCP/health group (adjusted OR = 4.39, 95% CI: 1.25, 15.35). The NCCP group had lower odds for better mental quality of life scores than the CCP group (adjusted OR = 0.90, 95% CI: 0.87, 0.94). We did not find any evidence that indicated the odds of severe or moderate anxiety had been increased in NCCP significantly. Furthermore, the NCCP group did not worsen the physical quality of life. None of the interaction forms entered into the multivariable model were significant. Moreover, there was no multicollinearity in the independent variables.

**Table 3:** Univariable and multivariable logistic regression for assessing the association between pain source (NCCP^b^ and CCP^a^ or healthy) with demographic variables, depression, anxiety, and quality of life (*n* = 210).

Variables	Univariable		Multivariable	
	OR (95% CI)	*p*-value	AOR (95% CI)	*p*-value
Age (year)	1.03 (0.98, 1.08)	0.163*	1.04 (0.97, 1.10)	0.213
Sex				
Female	0.75 (0.42, 1.34)	0.342	–	–
Male	1	1	–	–
Job				
Unemployed/housewife	0.66 (0.36, 1.21)	0.184*	0.56 (0.24, 1.32)	0.190
Employed	1	1	1	1
Depression				
Severe/moderate	14.06 (5.30, 40.23)	<0.001*	4.39 (1.25, 15.35)	0.020**
No/mild	1	1	1	1
Anxiety				
Severe/moderate	8.83 (4.35, 17.88)	<0.001*	1.83 (0.71, 4.66)	0.205
Minimal/mild	1	1	1	1
Quality of life				
Physical	0.92 (0.89, 0.95)	<0.001*	1.02 (0.98, 1.07)	0.272
Mental health	0.90 (0.87, 0.92)	<0.001*	0.90 (0.87, 0.94)	<0.001**

a CCP, cardiac chest pain.

b NCCP, non-cardiac chest pain.

*Variables with a *p-*value of ≤ 0.2 and entered into the multivariable model.** Statistical significance: *p*-value < 0.05.

## Discussion

In the present study, patients with NCCP had a higher chance of having moderate-to-severe depression and a lower quality of life than CCP or healthy people. The median score of depression and anxiety in people with NCCP was higher than in patients with CCP. These scores were higher in CCP compared to the control group.

In line with our study, a study in İzmir, Turkey, between 2015 and 2018 on patients aged 13–18 with unexplained chest pain showed that there was a significant association between unexplained chest pain with depression and impaired emotions (25). In Southeast Sweden, patients with NCCP and a history of cardiovascular disease (CD), compared to patients without CD had a poorer health-related quality of life (3). These results are similar to the present study, in which people with NCCP are associated with lower quality of life scores. Our findings are similar to those of Alkhatatbeh et al., who observed that anxiety and depression scores were higher in subjects with NCCP than healthy controls (26).

In contrast with the present study, a study in Hong Kong and Wuhan from 2004 to 2005 reported that the quality of life and psychological impact in patients with NCCP were not different from CCP (27). In another study conducted in Niš, Serbia, although the patients with NCCP had no associated psychiatric disorder, coronary patients were more depressed and hostile (28). These results may be due to differences in the study population and the questionnaires used.

People with NCCP may worry about the origin of symptoms. They may attribute their chest pain not only to the heart but also to other diagnoses, which can further lessen their quality of life (3). Anxiety and depression were supposed to be possible causes of NCCP (26). The coexistence of psychological disorders with NCCP (a pathophysiological mechanism) is very important (29). Some psychopharmacological treatments have been suggested to manage NCCP (30). Patients suffering from NCCP may have obsessional thoughts about the disease. They tend to have disastrous interpretations of their bodily sensations. Therefore, psychological treatments can help them (31).

Some limitations of the current study are the convenient sampling method and the focus on people over 45 years old. We had to use the conventional method to select healthy and chest pain groups. One of the other limitations was self-report which is subject to recall bias and potential inaccuracies in participants’ responses. The study was conducted in Shahrekord, Iran, which may restrict the generalizability of the findings to other populations or geographic locations.

The NCCP group had higher depression and anxiety and lower quality of life scores compared to the CCP and healthy groups. NCCP people had a higher chance for higher depression and lower mental quality of life. CCP participants had higher depression and anxiety scores and lower quality of life compared to healthy individuals. Therefore, it seems the cooperation of psychiatrists with other specialists is necessary to enhance patients’ health conditions and quality of life.

## Data availability statement

The raw data supporting the conclusions of this article will be made available by the authors, without undue reservation.

## Ethics statement

The studies involving humans were approved by the study was conducted in accordance with the Declaration of Helsinki, and approved by the Ethics Committee of Shahrekord University of Medical Sciences (IR.SKUMS.REC.1398.032). The studies were conducted in accordance with the local legislation and institutional requirements. Written informed consent for participation in this study was provided by the participants' legal guardians/next of kin.

## Author contributions

EZ: Conceptualization, Writing – original draft, Validation. ZS: Data curation, Investigation, Writing – review & editing. SK: Formal analysis, Methodology, Software, Writing – original draft. ST: Formal analysis, Methodology, Software, Writing – original draft, Conceptualization, Funding acquisition, Writing – review & editing.
